# 4-Sulfamoylanilinium perchlorate

**DOI:** 10.1107/S1600536813017972

**Published:** 2013-07-10

**Authors:** R. Anitha, S. Athimoolam, M. Gunasekaran, B. Sridhar

**Affiliations:** a Department of Physics, Regional centre of Anna University, Tirunelveli Region, Tirunelveli 627 007, India; b Department of Physics, University College of Engineering, Nagercoil, Anna University, Tirunelveli Region, Nagercoil 629 004, India; c Laboratory of X-ray Crystallography, Indian Institute of Chemical Technology, Hyderabad 500 607, India

## Abstract

In the crystal of the title salt, C_6_H_9_N_2_O_2_S^+^·ClO_4_^−^, the components are linked by N—H⋯O hydrogen bonds, forming a three-dimensional network. The cations are connected along *a* and *b* axes, leading to linear and zigzag *C*(3) and *C*(8) chain motifs, respectively. A cation–anion inter­action along the *c* axis leads to a *C*_2_^2^(12) chain motif. *R*_3_^3^(18) and *R*_3_^3^(20) ring motifs are observed as cation–anion-type inter­actions. These hydrogen-bonding ring and chain motifs are localized at *z* = 0 or 1, leading to alternate hydro­philic and hydro­phobic regions along the *c* axis as a result of the stacking of anions and the aromatic cationic parts.

## Related literature

For the first use of sulfanilamide, see: Buttle *et al.* (1936 ▶). For related structures, see: Ravikumar *et al.* (2013 ▶); Pandiarajan *et al.* (2011 ▶); Topacli & Kesimli (2001 ▶). For graph-set motifs, see Etter *et al.* (1990 ▶).
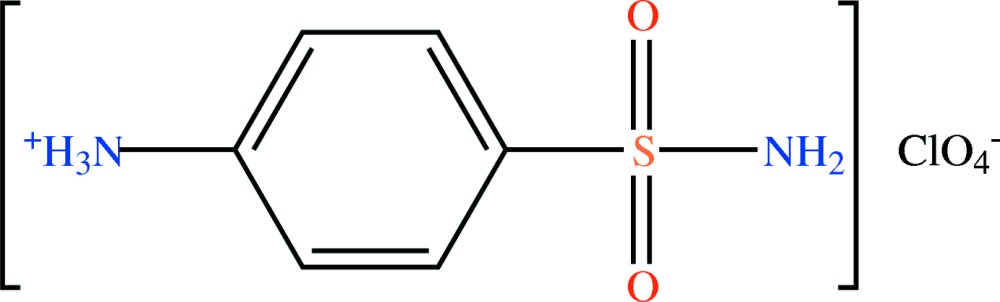


## Experimental

### 

#### Crystal data

C_6_H_9_N_2_O_2_S^+^·ClO_4_^−^
*M* *_r_* = 272.66
Monoclinic, 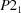

*a* = 4.9158 (10) Å
*b* = 10.514 (2) Å
*c* = 9.814 (2) Å
β = 93.716 (3)°
*V* = 506.15 (18) Å^3^
*Z* = 2
Mo *K*α radiation
μ = 0.60 mm^−1^
*T* = 293 K
0.24 × 0.16 × 0.12 mm

#### Data collection

Bruker SMART APEX CCD area-detector diffractometer
5796 measured reflections
2367 independent reflections
2361 reflections with *I* > 2σ(*I*)
*R*_int_ = 0.023

#### Refinement

*R*[*F* ^2^ > 2σ(*F* ^2^)] = 0.022
*wR*(*F* ^2^) = 0.061
*S* = 1.08
2367 reflections
166 parameters
5 restraints
H atoms treated by a mixture of independent and constrained refinement
Δρ_max_ = 0.31 e Å^−3^
Δρ_min_ = −0.26 e Å^−3^
Absolute structure: Flack (1983 ▶), 1287 Friedel pairs
Flack parameter: 0.00 (4)

### 

Data collection: *SMART* (Bruker, 2001 ▶); cell refinement: *SAINT* (Bruker, 2001 ▶); data reduction: *SAINT*; program(s) used to solve structure: *SHELXTL/PC* (Sheldrick, 2008 ▶); program(s) used to refine structure: *SHELXTL/PC*; molecular graphics: *PLATON* (Spek, 2009 ▶); software used to prepare material for publication: *SHELXTL/PC*.

## Supplementary Material

Crystal structure: contains datablock(s) global, I. DOI: 10.1107/S1600536813017972/ng5332sup1.cif

Structure factors: contains datablock(s) I. DOI: 10.1107/S1600536813017972/ng5332Isup2.hkl

Additional supplementary materials:  crystallographic information; 3D view; checkCIF report

## Figures and Tables

**Table 1:** Hydrogen-bond geometry (Å, °)

*D*—H⋯*A*	*D*—H	H⋯*A*	*D*⋯*A*	*D*—H⋯*A*
N1—H1*A*⋯O1^i^	0.88 (1)	2.12 (1)	2.953 (2)	160 (3)
N1—H1*B*⋯O6^ii^	0.88 (3)	2.30 (3)	3.086 (2)	149 (2)
N2—H2*A*⋯O5^i^	0.88 (1)	2.19 (1)	3.044 (2)	164 (3)
N2—H2*B*⋯O2^iii^	0.88 (1)	2.16 (2)	2.876 (2)	139 (2)
N2—H2*C*⋯O4^iv^	0.88 (1)	2.30 (2)	2.858 (2)	122 (2)
N2—H2*C*⋯O5	0.88 (1)	2.37 (2)	3.058 (2)	136 (2)

Symmetry codes: (i) 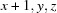; (ii) 
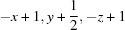
; (iii) 
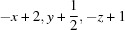
; (iv) 
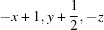
.

**Fig. 1.**
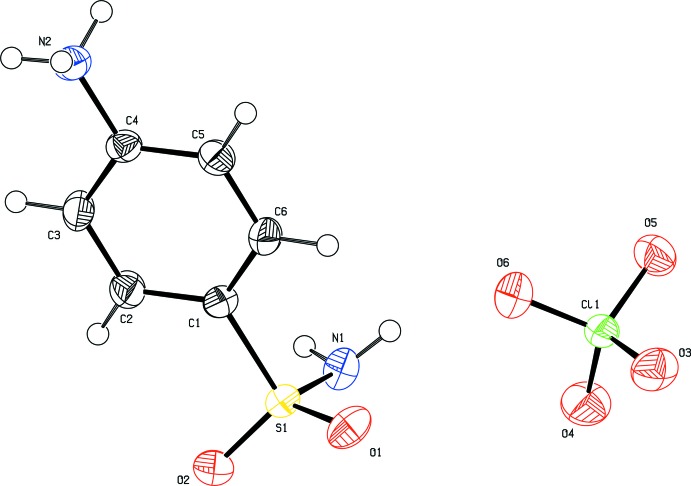
The title molecule with the atom numbering scheme. The displacement ellipsoids are shown at the 50% probability level.

**Fig. 2.**
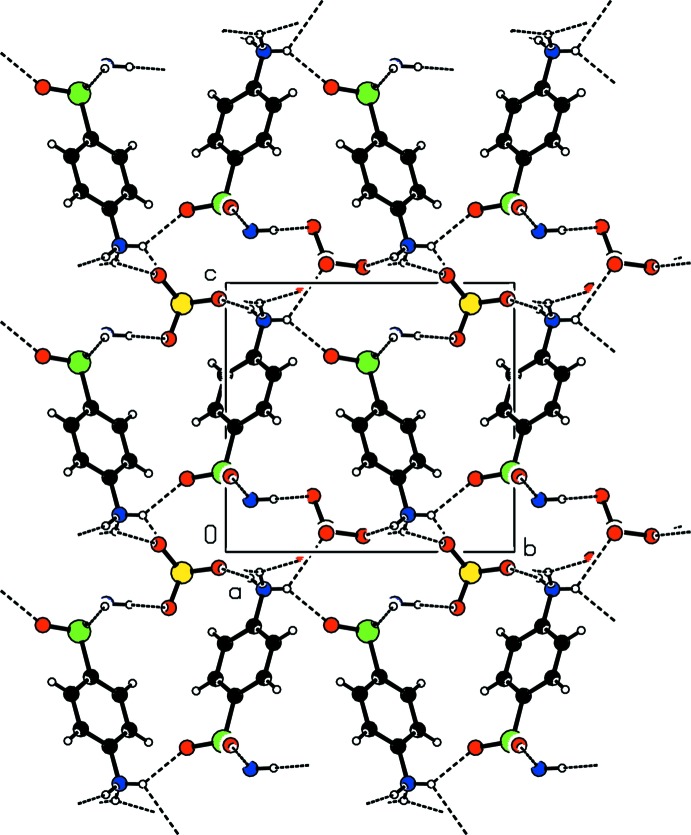
Packing diagram of the title compound viewed down the aaxis. The H-bonds are shown as dashed lines.

## supplementary crystallographic information

### Comment

Sulfa drugs, mostly the derivatives of sulfanilamide, have been an integral part of our medical history. They were the first effective chemotherapeutic agents to be widely used for the treatment of bacterial infection in humans and animals (Topacli & Kesimli, 2001). The uses of Sulfanilamide, was started during 1936 (Buttle *et al.*, 1936) and the grandparent of the sulfonamide family of drugs that are still in use today. The nitrate and sulfate complexes of sulfanilamide (Pandiarajan *et al.*, 2011; Ravikumar *et al.*, 2013) were already reported. In continuation of our interest on the sulfanilamide complexes, the synthesis of the title compound and its title structure,bis(4-sulfamoylanilinium) sulfate, is described here. In the title structure, a protonated sulfomylanilinium cation and a perchlorate anion constitute the asymmetric part (Fig. 1). The protonation on the one of N sites is confirmed from C—N bond distance. The geometrical parameters of the cation are in agreement with the reported sulfomylanilinium structures in 4-sulfomylanilinium nitrate (Pandiarajan *et al.*, 2011) and Bis(4-sulfomylanilinium) sulfate (Ravikumar *et al.*, 2013).

The crystal structure is stabilized through intricate three dimensional hydrogen bonding network formed through N—H···O interactions (Table 1; Fig. 2). All the hydrogen atoms attached to both the nitrogen atoms of the cation is involved in the hydrogen bonding interactions as donors. All the oxygen atoms in the cation and anion, except the O3 atom present in the anion, are acting as acceptor atoms and involved in the hydrogen bonding interactions. One of the N—H···O hydrogen bonds is observed to be bifurcated hydrogen bond, with one donor hydrogen (Table 1): N2—H3N···O4 (-*x* + 1, *y* + 1/2, -*z*) and N2—H3N···O5. Among the hydrogen bonds, two are cation-cation type and other four hydrogen bonds are cation-anion type. In the cation-cation type, N1—H2N···O1(1 + *x*, *y*, *z*) is making a chain C(3) motif extending along *a*-axis of the unit cell. Another N2—H5N···O2(2 - *x*, 1/2 + *y*, 1 - *z*) hydrogen bond is connecting the cations along *b*-axis of the unit cell through zigzag chain C(8) motifs. Thus cations are connected directly only along *a* & *b*-axes. Whereas along *c*-axis the interactions are cation-anion type, *i.e.*, the cations and anions are connected through N1—H1N···O6(-*x* + 1, *y* + 1/2, -*z* + 1) and N2—H3N···O4(-*x* + 1, *y* + 1/2, -*z*) hydrogen bonds leading to chain C_2_2^(^12) motif.

Further, cations and anions are connected through N1—H2N···O1(1 + *x*, *y*, *z*), N2—H3N···O5 and N2—H4N···O5(1 + *x*, *y*, *z*) leading to a ring *R*_3_^3^(18) motifs. Another ring *R*_3_^3^(20) motif is formed through N1—H1N···O6(-*x* + 1, *y* + 1/2, -*z* + 1), N2—H4N···O5(1 + *x*, *y*, *z*) and N2—H5N···O2(-*x* + 2, *y* + 1/2, -*z* + 1) hydrogen bonds. These ring *R*_3_^3^(20) motifs are arranged adjacently and making a chain C_3_^3^(10) motif extending along *b*-axis of the unit cell. These hydrogen bonding ring and chain motifs are localized at *z* = 0 or *z* = 1 leading to alternate hydrophilic and hydrophobic regions along *c* axis as a result of the stacking of anions and the aromatic cationic parts.

### Experimental

The synthesis of the title compound was carried out by heating of the mixture of sulphanilamide (1.7 g) and perchloric acid (0.5 ml of 98%)in water with the stoichiometric ratio of 1:1 (at 60°C) under reflux for 1 h. Colourless needle type crystals of the title compound suitable for single-crystal X-ray analysis with the approximate size of 1.8 cm τimes 0.6 cm τimes 0.4 cm were obtained by slow evaporation at room temperature. The measured sample was cut from a bigger crystal. Caution: Although no problems were encountered in this work, perchlorate compounds are potentially explosive. They should be prepared in small amounts and handled with care.

### Refinement

All the H atoms except the atoms involved in hydrogen bonds were positioned geometrically and refined using a riding model, with C—H = 0.93 Å and *U*_iso_(H) = 1.2 *U*_eq_ (parent atom). H atoms involved in hydrogen bonds were located from differential fourier map and refined isotropically with the distance restraint (*DFIX*) for appropriate distance (0.88 (1) Å). From the measured 5796 total reflections, 1287 are Friedel opposites.

### Crystal data

**Table:**

C_6_H_9_N_2_O_2_S^+^·ClO_4_^−^	*F*(000) = 280
*M**_r_* = 272.66	*D*_x_ = 1.789 Mg m^−^^3^
Monoclinic, *P*2_1_	Mo *K*α radiation, λ = 0.71073 Å
Hall symbol: P 2yb	Cell parameters from 2412 reflections
*a* = 4.9158 (10) Å	θ = 2.5–24.7°
*b* = 10.514 (2) Å	µ = 0.60 mm^−^^1^
*c* = 9.814 (2) Å	*T* = 293 K
β = 93.716 (3)°	Needle, colourless
*V* = 506.15 (18) Å^3^	0.24 × 0.16 × 0.12 mm
*Z* = 2	

### Data collection

**Table:**

Bruker SMART APEX CCD area-detector diffractometer	2361 reflections with *I* > 2σ(*I*)
Radiation source: fine-focus sealed tube	*R*_int_ = 0.023
Graphite monochromator	θ_max_ = 28.0°, θ_min_ = 2.1°
ω scans	*h* = −6→6
5796 measured reflections	*k* = −13→13
2367 independent reflections	*l* = −12→12

### Refinement

**Table:**

Refinement on *F*^2^	Hydrogen site location: inferred from neighbouring sites
Least-squares matrix: full	H atoms treated by a mixture of independent and constrained refinement
*R*[*F*^2^ > 2σ(*F*^2^)] = 0.022	*w* = 1/[σ^2^(*F*_o_^2^) + (0.0315*P*)^2^ + 0.1129*P*] where *P* = (*F*_o_^2^ + 2*F*_c_^2^)/3
*wR*(*F*^2^) = 0.061	(Δ/σ)_max_ = 0.001
*S* = 1.08	Δρ_max_ = 0.31 e Å^−^^3^
2367 reflections	Δρ_min_ = −0.26 e Å^−^^3^
166 parameters	Extinction correction: *SHELXTL/PC* (Sheldrick, 2008), Fc^*^=kFc[1+0.001xFc^2^λ^3^/sin(2θ)]^-1/4^
5 restraints	Extinction coefficient: 0.189 (7)
Primary atom site location: structure-invariant direct methods	Absolute structure: Flack (1983), 1287 Friedel pairs
Secondary atom site location: difference Fourier map	Flack parameter: 0.00 (4)

### Special details

**Table:**

Geometry. All e.s.d.'s (except the e.s.d. in the dihedral angle between two l.s. planes) are estimated using the full covariance matrix. The cell e.s.d.'s are taken into account individually in the estimation of e.s.d.'s in distances, angles and torsion angles; correlations between e.s.d.'s in cell parameters are only used when they are defined by crystal symmetry. An approximate (isotropic) treatment of cell e.s.d.'s is used for estimating e.s.d.'s involving l.s. planes.
Refinement. Refinement of *F*^2^ against ALL reflections. The weighted *R*-factor *wR* and goodness of fit *S* are based on *F*^2^, conventional *R*-factors *R* are based on *F*, with *F* set to zero for negative *F*^2^. The threshold expression of *F*^2^ > σ(*F*^2^) is used only for calculating *R*-factors(gt) *etc*. and is not relevant to the choice of reflections for refinement. *R*-factors based on *F*^2^ are statistically about twice as large as those based on *F*, and *R*- factors based on ALL data will be even larger.

### Fractional atomic coordinates and isotropic or equivalent isotropic displacement parameters (Å^2^)

**Table:**

	*x*	*y*	*z*	*U*_iso_*/*U*_eq_	
C1	0.8057 (3)	0.53210 (15)	0.53535 (15)	0.0244 (3)	
C2	1.0030 (3)	0.46394 (16)	0.47189 (16)	0.0295 (3)	
H2	1.0919	0.3964	0.5169	0.035*	
C3	1.0672 (3)	0.49695 (19)	0.34095 (16)	0.0293 (3)	
H3	1.1998	0.4523	0.2973	0.035*	
C4	0.9310 (3)	0.59698 (15)	0.27676 (15)	0.0235 (3)	
C5	0.7316 (3)	0.66524 (16)	0.33789 (17)	0.0281 (3)	
H5	0.6419	0.7320	0.2920	0.034*	
C6	0.6676 (3)	0.63237 (17)	0.46891 (17)	0.0294 (3)	
H6	0.5338	0.6768	0.5119	0.035*	
N1	0.9084 (3)	0.58560 (16)	0.80644 (15)	0.0315 (3)	
N2	0.9994 (3)	0.63135 (15)	0.13833 (15)	0.0279 (3)	
O1	0.4527 (2)	0.51671 (14)	0.71892 (14)	0.0380 (3)	
O2	0.8370 (3)	0.36510 (13)	0.72799 (14)	0.0385 (3)	
H1A	1.0834 (17)	0.578 (3)	0.797 (3)	0.070 (9)*	
H1B	0.847 (5)	0.665 (3)	0.803 (3)	0.047 (7)*	
H2A	1.120 (4)	0.584 (2)	0.100 (3)	0.055 (7)*	
H2B	1.052 (5)	0.7108 (9)	0.134 (3)	0.053 (8)*	
H2C	0.863 (4)	0.617 (3)	0.078 (2)	0.048 (7)*	
Cl1	0.58672 (7)	0.34769 (3)	0.07752 (4)	0.02621 (11)	
O3	0.8773 (2)	0.34793 (17)	0.06980 (15)	0.0409 (3)	
O4	0.4686 (3)	0.26701 (16)	−0.02835 (16)	0.0477 (4)	
O5	0.4874 (3)	0.47611 (13)	0.05900 (15)	0.0399 (3)	
O6	0.5169 (3)	0.30181 (14)	0.20823 (15)	0.0445 (3)	
S1	0.73500 (7)	0.49156 (3)	0.70441 (4)	0.02528 (11)	

### Atomic displacement parameters (Å^2^)

**Table:**

	*U*^11^	*U*^22^	*U*^33^	*U*^12^	*U*^13^	*U*^23^
C1	0.0265 (7)	0.0256 (7)	0.0217 (7)	−0.0006 (6)	0.0055 (5)	0.0017 (5)
C2	0.0328 (7)	0.0291 (8)	0.0268 (7)	0.0085 (6)	0.0034 (6)	0.0022 (6)
C3	0.0318 (7)	0.0310 (8)	0.0258 (7)	0.0071 (7)	0.0066 (5)	−0.0021 (6)
C4	0.0246 (6)	0.0244 (7)	0.0217 (6)	−0.0033 (5)	0.0026 (5)	0.0002 (5)
C5	0.0295 (8)	0.0260 (8)	0.0289 (7)	0.0051 (6)	0.0039 (6)	0.0046 (6)
C6	0.0286 (7)	0.0294 (8)	0.0311 (8)	0.0067 (6)	0.0091 (6)	0.0020 (7)
N1	0.0303 (7)	0.0361 (8)	0.0285 (7)	0.0003 (6)	0.0048 (6)	−0.0044 (6)
N2	0.0309 (7)	0.0293 (7)	0.0239 (6)	−0.0016 (6)	0.0045 (5)	0.0020 (6)
O1	0.0264 (6)	0.0523 (9)	0.0362 (6)	−0.0018 (5)	0.0090 (5)	0.0027 (6)
O2	0.0532 (8)	0.0281 (7)	0.0355 (6)	0.0052 (6)	0.0131 (5)	0.0080 (5)
Cl1	0.02693 (18)	0.02327 (17)	0.02901 (19)	−0.00278 (13)	0.00626 (12)	−0.00363 (14)
O3	0.0255 (6)	0.0483 (7)	0.0495 (7)	0.0003 (6)	0.0069 (5)	−0.0073 (7)
O4	0.0489 (8)	0.0478 (8)	0.0466 (9)	−0.0168 (7)	0.0033 (7)	−0.0193 (7)
O5	0.0413 (7)	0.0271 (7)	0.0522 (8)	0.0048 (6)	0.0092 (6)	0.0027 (6)
O6	0.0560 (8)	0.0424 (8)	0.0366 (7)	−0.0042 (6)	0.0155 (6)	0.0060 (6)
S1	0.02663 (18)	0.02599 (19)	0.02388 (17)	0.00010 (14)	0.00668 (12)	0.00191 (14)

### Geometric parameters (Å, º)

**Table:**

C1—C2	1.386 (2)	N1—S1	1.6113 (16)
C1—C6	1.393 (2)	N1—H1A	0.875 (5)
C1—S1	1.7692 (15)	N1—H1B	0.88 (3)
C2—C3	1.387 (2)	N2—H2A	0.877 (5)
C2—H2	0.9300	N2—H2B	0.876 (5)
C3—C4	1.377 (2)	N2—H2C	0.876 (5)
C3—H3	0.9300	O1—S1	1.4289 (13)
C4—C5	1.383 (2)	O2—S1	1.4345 (14)
C4—N2	1.466 (2)	Cl1—O6	1.4328 (14)
C5—C6	1.387 (2)	Cl1—O4	1.4345 (14)
C5—H5	0.9300	Cl1—O3	1.4355 (12)
C6—H6	0.9300	Cl1—O5	1.4432 (14)
			
C2—C1—C6	121.01 (14)	H1A—N1—H1B	115 (3)
C2—C1—S1	118.86 (12)	C4—N2—H2A	117.2 (19)
C6—C1—S1	120.12 (12)	C4—N2—H2B	111.4 (18)
C1—C2—C3	119.67 (15)	H2A—N2—H2B	108 (3)
C1—C2—H2	120.2	C4—N2—H2C	111.9 (18)
C3—C2—H2	120.2	H2A—N2—H2C	97 (3)
C4—C3—C2	118.80 (15)	H2B—N2—H2C	110 (3)
C4—C3—H3	120.6	O6—Cl1—O4	109.89 (10)
C2—C3—H3	120.6	O6—Cl1—O3	110.16 (9)
C3—C4—C5	122.36 (15)	O4—Cl1—O3	108.68 (9)
C3—C4—N2	118.57 (14)	O6—Cl1—O5	109.20 (9)
C5—C4—N2	119.06 (14)	O4—Cl1—O5	110.13 (10)
C4—C5—C6	118.85 (14)	O3—Cl1—O5	108.75 (9)
C4—C5—H5	120.6	O1—S1—O2	119.15 (9)
C6—C5—H5	120.6	O1—S1—N1	107.62 (8)
C5—C6—C1	119.31 (14)	O2—S1—N1	107.65 (9)
C5—C6—H6	120.3	O1—S1—C1	107.46 (7)
C1—C6—H6	120.3	O2—S1—C1	106.66 (7)
S1—N1—H1A	111 (2)	N1—S1—C1	107.86 (8)
S1—N1—H1B	112.7 (17)		
			
C6—C1—C2—C3	0.9 (3)	C2—C1—C6—C5	−0.8 (2)
S1—C1—C2—C3	−177.99 (13)	S1—C1—C6—C5	178.07 (13)
C1—C2—C3—C4	−0.3 (3)	C2—C1—S1—O1	−147.96 (13)
C2—C3—C4—C5	−0.3 (3)	C6—C1—S1—O1	33.10 (16)
C2—C3—C4—N2	−179.92 (15)	C2—C1—S1—O2	−19.12 (16)
C3—C4—C5—C6	0.4 (2)	C6—C1—S1—O2	161.93 (14)
N2—C4—C5—C6	−179.99 (15)	C2—C1—S1—N1	96.28 (15)
C4—C5—C6—C1	0.2 (2)	C6—C1—S1—N1	−82.67 (15)

### Hydrogen-bond geometry (Å, º)

**Table:**

*D*—H···*A*	*D*—H	H···*A*	*D*···*A*	*D*—H···*A*
N1—H1*A*···O1^i^	0.88 (1)	2.12 (1)	2.953 (2)	160 (3)
N1—H1*B*···O6^ii^	0.88 (3)	2.30 (3)	3.086 (2)	149 (2)
N2—H2*A*···O5^i^	0.88 (1)	2.19 (1)	3.044 (2)	164 (3)
N2—H2*B*···O2^iii^	0.88 (1)	2.16 (2)	2.876 (2)	139 (2)
N2—H2*C*···O4^iv^	0.88 (1)	2.30 (2)	2.858 (2)	122 (2)
N2—H2*C*···O5	0.88 (1)	2.37 (2)	3.058 (2)	136 (2)

Symmetry codes: (i) *x*+1, *y*, *z*; (ii) −*x*+1, *y*+1/2, −*z*+1; (iii) −*x*+2, *y*+1/2, −*z*+1; (iv) −*x*+1, *y*+1/2, −*z*.

## References

[bb1] Bruker (2001). *SAINT* and *SMART* Bruker AXS Inc., Madison, Wisconsin, USA.

[bb2] Buttle, G. A. H., Grey, W. H. & Stephenson, D. (1936). *Lancet*, **1**, 1286–1290.

[bb3] Etter, M. C., MacDonald, J. C. & Bernstein, J. (1990). *Acta Cryst.* B**46**, 256–262. 10.1107/s0108768189012929 2344397

[bb4] Flack, H. D. (1983). *Acta Cryst.* A**39**, 876–881.

[bb5] Pandiarajan, S., Balasubramanian, S., Ravikumar, B. & Athimoolam, S. (2011). *Acta Cryst.* E**67**, o2788. 10.1107/S1600536811038827 PMC3201237 22058822

[bb6] Ravikumar, B., Pandiarajan, S. & Athimoolam, S. (2013). *Acta Cryst.* E**69**, o596. 10.1107/S1600536813007216 PMC3629637 23634124

[bb7] Sheldrick, G. M. (2008). *Acta Cryst.* A**64**, 112–122. 10.1107/S0108767307043930 18156677

[bb8] Spek, A. L. (2009). *Acta Cryst.* D**65**, 148–155. 10.1107/S090744490804362X PMC2631630 19171970

[bb9] Topacli, A. & Kesimli, B. (2001). *Sepctrosc. Lett.* **34**, 513–526.

